# The Roles and Responsibilities of Community Pharmacists Supporting Older People with Palliative Care Needs: A Rapid Review of the Literature

**DOI:** 10.3390/pharmacy8030143

**Published:** 2020-08-12

**Authors:** Paul Tait, Amal Chakraborty, Jennifer Tieman

**Affiliations:** 1 Southern Adelaide Palliative Services, Flinders Medical Centre, SA Health, Bedford Park, SA 5042, Australia; 2 Research Centre for Palliative Care, Death and Dying, College of Nursing and Health Sciences, Flinders University, Bedford Park, SA 5042, Australia; amal.chakraborty@flinders.edu.au (A.C.); jennifer.tieman@flinders.edu.au (J.T.)

**Keywords:** palliative care, residential aged care, community pharmacist, medication review, multidisciplinary team

## Abstract

Globally, the number of older people requiring appropriate and safe management of medicines is growing. This review aimed to identify the roles and responsibilities of pharmacists supporting older people living in a community setting with their palliative care needs and to synthesise key themes emerging from the data, as well as any gaps in knowledge. The literature search included Medline (Ovid), Scopus, and Cinahl (Ebsco) databases. An English language limit was applied. The search included all international articles and any date of publication. Data were synthesised utilizing a systematic text condensation technique and presented according to Theme, Domain, and Meaning Units. Fourteen studies met the inclusion criteria. Selected papers predominantly focused on care provided by the pharmacists supporting people receiving residential aged care services. Clinical review, supply of medicines, and clinical governance were identified as key pharmacist roles. Pharmacists’ communication skills, personal behavioural approach, and positive attitude emerged as supportive characteristics for effective person-centered care. Minimal, or no information, were available related to pharmacists located in general medical practices and in Aboriginal health services sector, respectively. The multifaceted role of pharmacists presents an opportunity to provide comprehensive health care for older populations at the end of their life.

## 1. Introduction

As the population ages, the number of older Australians with palliative care needs is increasing [1]. Multimorbidity is common and this typically contributes to significant polypharmacy [2]. While polypharmacy can be appropriate, there is considerable evidence for its ability to cause harm, which is preventable [3]. Clearly, appropriate and safe management of medicines is an important aspect of care for older people [4]. Yet, there are several points of weakness in the medication management process which can contribute to poor outcomes [5,6]. In partnership with the multidisciplinary team, community pharmacists are ideal people to facilitate good medicines management for older people, built upon their clinical expertise and existing relationships with people they service, their carers, and the broader healthcare workforce [7,8]. The 2019 report *“PHARMACISTS IN 2023: For patients, for our profession, for Australia’s health system”* describes the broad remit of Australian pharmacists. It outlines some of the non-dispensing roles that community pharmacists have with care teams such as advising on medicine management, medicine safety, and the rational use of medicines in a cost-effective manner [9].

While changes in an older person’s condition can contribute to multiple hospital admissions, the last 12 months of an older person’s life is spent predominantly in the community [10,11]. They may receive care through:

- Home Care (HC) services—where the person receives care in their home dwelling; or
- Residential Aged Care (RAC) services—where the individual is provided care within a Residential Aged Care Home (RACH).

In Australia, a multidisciplinary approach to care is dependent on a range of Non-Government Organisations (NGO) working together, including general medical practices, aged care providers, Aboriginal health services, and dispensing pharmacy services. NGOs predominantly deliver care that is subsidised using National funding levers, including the Medicare Benefits Schedule (MBS), 6th Community Pharmacy Agreement (6CPA), Pharmaceutical Benefits Scheme (PBS), and Home Care Packages Program (HCPP). Within this complex, multi-faceted Australian healthcare system, rational use of medication management services for older people is provided by the primary healthcare services and aged care organisations collaboratively via referrals between pharmacists and general medical practitioners [12].

Although structures exist to facilitate pharmacist involvement in care, we are unaware of any extensive research discussing the full spectrum of their roles and responsibilities, specifically relating to the care of older people receiving HC or RAC toward the end of their life [13]. This rapid review aimed to identify international published literature that describes the roles and responsibilities of community pharmacists supporting older people receiving HC or RAC with their palliative care needs, to synthesise key themes emerging from the data, as well as identify any gaps in knowledge.

## 2. Materials and Methods

This rapid review applied a streamlined systematic review method [14,15]. Scientific peer reviewed journal articles were retrieved through searching in electronic databases. The search strategy was developed and tested in Medline (Ovid) with the help of a Health Librarian (SH). Broad text words and MeSH headings were used with relevance to palliative care, aged care in a community setting, and the role of a pharmacist. An English language limit was applied due to time and resource constraints. No date limit was applied. Furthermore, searches included articles from any country. Once the search was finalised and run in Medline (Ovid), it was then translated and run in Scopus and Cinahl (Ebsco) on the 23 July 2019.

The full search strategies for each database are detailed in online only Supplementary Tables S1–S3. The search results for each database were uploaded to Endnote X9.2 reference management software and deduplicated [16]. Journal articles were then imported into the web-based software program Covidence for screening and data extraction [17].

### 2.1. Inclusion Criteria

Criteria for inclusion weref developed—these are summarised in Table 1.

Two reviewers (SH and PT) independently assessed titles and abstracts against the priori inclusion criteria outlined in Table 1. Where eligibility was unclear based on the title and abstract screening, the full text article was retrieved and assessed. Any disagreements on eligibility for inclusion were resolved by discussions with a third reviewer (JT), if necessary.

The full-text articles identified from the title and abstract screening were independently assessed by two reviewers (AC and PT), using the inclusion criteria before selecting for final data extraction and synthesis. Reference lists of the included studies were not examined to identify additional articles. A range of published literature were included, such as papers of experimental and quasi-experimental primary studies, review papers, program evaluation reports, expert commentaries, and surveys.

Studies were excluded if they lacked discussion of the role of the pharmacist, had no specific focus on older people, or were describing care in an acute hospital care setting (including discharge planning).

### 2.2. Data Extraction

A data extraction tool was developed using Microsoft Excel and tested with three randomly selected articles. Two researchers (PT and AC) extracted the following data: (1) study characteristics, (2) summary description, and (3) Data Elements. Study characteristics pertained to author, study year, study design, study setting, country, and level of evidence. The assessment of “level of evidence” employed an adaptation of the Johns Hopkins Model of Evidence-Based Practice [18]. The levels of evidence in papers were organised into five categories (Table 2), where Level I represents the strongest quality of the evidence [18]. Summary description included brief information on what the study contains. Data Elements included the roles and responsibilities of community pharmacists identified in the full text screening.

### 2.3. Synthesis of Data

Synthesis of data was conducted in sequential steps utilizing a systematic text condensation technique [19].

Two researchers (AC and PT) independently read each of the full text papers to establish a preliminary list of Themes. The researchers then reviewed each of the included papers, line by line, to identify Data Elements. A Data Element was defined as a text fragment that described a certain idea (e.g., pharmacist providing medicines useful in symptom management for pain for a resident in aged care home). Common Data Elements were combined into a single Meaning Unit (e.g., supply of medicines to a RACH). Domains emerged out of the Data Elements through linking similar Meaning Units into groups (e.g., medicine supply). Domains were then mapped into relevant Themes.

Assignment of Data Elements into Meaning Units and Domains were performed independently by two researchers and the results were combined with consensus. An overview analysis of the coded Data Elements by individual researchers (AC and PT) determined that a high degree of inter- and intra-group thematic homogeneity existed, suggesting that the data could be combined with minimal bias.

## 3. Results

### 3.1. Literature Search, Screening, and Selection of Papers

**Figure 1:**
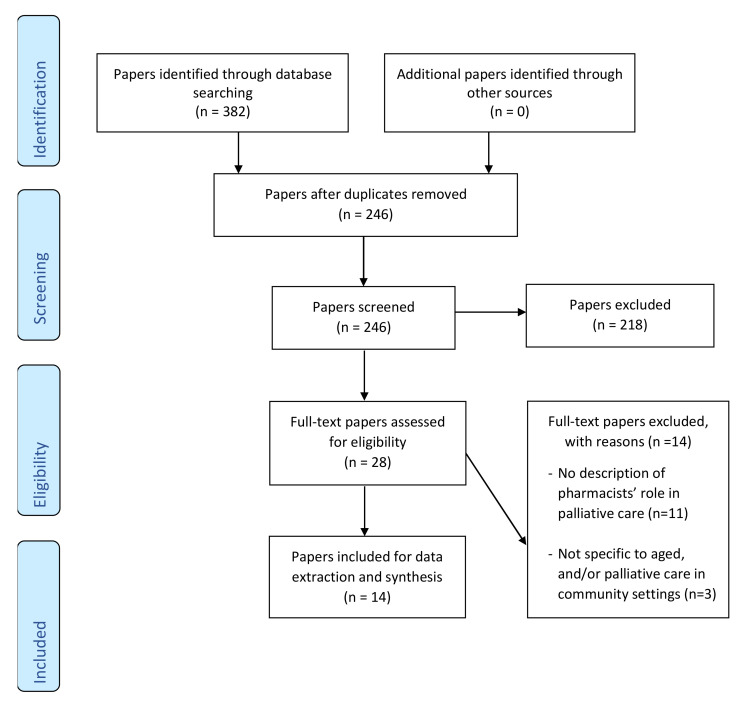
PRISMA Flow Diagram describing the paper selection process.

Figure 1 shows search results in a PRISMA (Preferred Reporting Items for Systematic Reviews and Meta-Analyses) Flow Diagram and the findings are reported following the PRISMA checklist (Supplementary Table S4). The electronic database search identified a total of 382 citations. After removing duplicates, the title and abstract screening of 246 citations identified 28 potential papers for full text review. Eligibility assessment resulted in 14 papers [20,21,22,23,24,25,26,27,28,29,30,31,32,33] meeting the inclusion criteria and being selected for final data extraction and synthesis.

### 3.2. Characteristics of the Selected Papers

Further characteristics of included papers are provided in Table 3. Of the 14 included papers, seven were conducted in the United States of America (USA) [21,26,27,28,30,31,32], four in Australia [22,23,25,33], two in the United Kingdom (UK) [20,29], and one in Canada [24]. Six of the 14 papers [20,21,22,26,30,31] described pharmacists’ role predominantly for people receiving RAC services. Three papers [23,24,27] discussed pharmacists’ role in caring for older people receiving HC services. The remaining five papers discussed care for people receiving either RAC or HC services [25,28,29,32,33].

Among the included papers, the levels of evidence varied [18]. Seven papers [20,22,23,25,26,32,33] were classified at the Level III evidence level. These included three papers [20,26,32] which systematically reviewed and synthesised best practice clinical interventions and four papers used observational qualitative research spanning across semi-structured interviews [22,23], focus groups [25], and surveys [33]. The remaining seven papers [21,24,27,28,29,30,31] were classified at the Level V evidence level. These included three expert commentaries [21,24,29], two case studies [27,28], one comprehensive literature review [31], and one [30] describing a pilot phase of a “quality improvement” intervention. The levels of evidence were comparable for papers focusing on receipt of either RAC or HC services.

### 3.3. Data Extraction

In total, 196 Data Elements were identified from the 14 selected papers (see Table 3). These were combined in 37 Meaning Units and 8 Domains. Three broad Themes relating to the roles and responsibilities of community pharmacists with older people living in the community were determined (see Table 4):

(1) Type of care delivery;
(2) Work context of the pharmacist; and
(3) Supportive professional and personal characteristics as soft skills.

#### 3.3.1. Theme One: Type of Care Delivery

The selected articles examined various pharmacist-led services for older people with palliative care needs. These included clinical review, supply of medicines, and contribution to clinical governance.

*Clinical review*

The bulk of the 14 papers discussed the pharmacist’s involvement in direct person-centred care involving a one-on-one clinical review. While this often involved the older person, some papers discussed inclusion of their carer [25,27,29]. The clinical reviews were conducted in people receiving both HC and RAC services. These included individualised medicines management such as medication reconciliation [23,26,27], recommendation of changes to medication doses [22,30], identifying medication related problems, and ensuring safety and appropriateness of prescribed medications [28,31,32]. Other activities described within a clinical review involved deprescribing of medicines that were no longer required, including analgesia and sedatives [20,21,27,30,31].

A number of key barriers to providing pharmacist-led clinical reviews were identified including: inadequate remuneration [22]; involvement of multiple prescribers [23]; poor processes for information sharing between providers [23]; unrealistic family expectations [22]; and poor health literacy among the population [25].

*Supply of medicines*

Responsibilities relating to supply of medicines featured in a few of the included papers, with references to people in receipt of HC and RAC services. This Theme comprised activities related to ordering and stocking of medicines [25]; dispensing [28]; delivery of medicines [28]; providing medicines information and counselling [21,25,29,33]; disposing unwanted medicines [27]; and provision of medicines in dose administration aids [27,28,29]. Some papers focused on the supply of medicines in the last days of life and the issues relating to poor access to subcutaneous medicines as the oral route is lost [25,33].

*Clinical governance*

Pharmacist roles also involved indirect care through broader engagement at the organisational level [22,23,28,31,32,33]. These papers focused on people receiving RAC services and discussed favourable organisation-wide changes—both clinical and financial—resulting from pharmacist involvement. Pharmacist advisors to a Medicines Advisory Committee (MAC) assisted in the development, promotion, monitoring, review, and evaluation of medication management policies, guidelines, and procedures and thus influenced the health and quality of life for all people cared for by the organisation. Other cited examples of pharmacists in indirect roles involved the provision of education to the nursing workforce around medicines and auditing of medication usage, resulting in cost savings [22,32]. Barriers to pharmacist involvement at the organizational level were inadequate remuneration.

#### 3.3.2. Theme Two: Work Setting of the Pharmacist

Ten of the papers described pharmacists working in a range of work settings, including Community Pharmacy, Residential Aged Care Homes (RACH), and General Medical Practice [20,22,23,25,27,28,30,31,32,33]. Each work setting offered different opportunities for the pharmacist to engage with the multidisciplinary workforce. These papers discussed a range of disciplines that pharmacists worked alongside, including general practitioners (GP), specialists in pain management and palliative care, allied health professionals, nurses, and medical administrators. No studies discussed the role of pharmacists within Aboriginal health services.

*Community Pharmacy*

Aside from their dispensing role, pharmacists working in community pharmacy also provide direct medication management support for those receiving HC services through informal connections with local GPs. Barriers to care are related to community pharmacies being geographically isolated from prescribers. Selected papers illustrated activities that maintain the pharmacist’s connection with the local healthcare teams, including:

- Real time liaison with GPs as part of case conferencing [22];
- Clarification of information relating to the prescription, including changes to the packing of dose administration aids [23]; and
- Anticipating which subcutaneous medicines to stock that are useful in managing symptoms expected in the last days of life [25].

These connections were led by the person’s acute needs and were often driven at an individual clinician level.

*Residential Aged Care Homes*

Many papers described the pharmacist’s clinical role or function of supply of medicines to the organization within a context of a formal arrangement or contract between the organisation providing RAC services and the individual pharmacist or pharmacy. As such, the role of the pharmacist within a multidisciplinary team was largely process driven, providing consistent care across the organisation, impacting on all people living in the organization. The clinical role of the pharmacist in this setting—such as the “medication review”—is performed in consultation with onsite nurses and GPs. In one paper, this role extended to communication and handover of medicines information at critical transitions of care such as admission to the RACH [26].

*General Medical Practice*

One study described how pharmacists working in a general medical practice setting improved timeliness and quality in how medication reviews were conducted [22]. Employing pharmacists within the general medical practice setting provided an opportunity to develop screening criteria for medicines prescribed by the GPs, such as checking medication lists for drug interactions, identifying duplication of therapy, and identifying problematic side effects; and facilitating external referral pathways. Pharmacists based at a general medical practice were also recognised as a resource for practice staff and community, with their timely provision of medicines information enabling effective coordination of home medication reviews for older people living in their home.

#### 3.3.3. Theme Three: Supportive Professional and Personal Characteristics as Soft Skills

Four [22,27,28,32] of the 14 papers highlighted the importance of having supportive professional and personal characteristics. These supportive characteristics demonstrate soft skills of pharmacists, such as communication skills, personal behavioural approach to other clinicians, and positive attitude for the pharmacist workforce towards effective person-centered care. The supportive characteristics identified in the review were categorised into two levels: (1) Soft skills in supporting person-centered care (the people they provide services for as well as their carers) and (2) Soft skills in working with clinician prescribers.

*Soft skills in supporting person-centered care*

Pharmacists advocate for and follow up on behalf of the people they provide services for (as well as their carers), ensuring better clinical outcomes. Examples of these skills include:

- Advocating with prescribers (e.g., GPs and specialists) to change medicines or doses and/or deprescribe medicines that maybe are unnecessary [30]; and
- Following-up with people after a home visit to monitor how changes to medicines are going and answer any medication-related questions [27].

*Soft skills in working with clinician prescribers*

Key skills of the community pharmacist workforce that enable building effective working relationships with a range of clinicians included creative communication and people skills. Examples of these skills are:

- Writing medication review recommendations as a “medication management plan” to make it more acceptable and relevant for GPs to provide feedback [22];
- Supporting and maintaining trusting relationships with a multidisciplinary team of practitioners [22];
- Demonstrating a positive and helpful attitude to medication prescribers and other clinicians [22];
- Communicating with medication prescribers in a clear and honest manner [22]; and
- Following-up with medication prescribers if no response to medication reviews outcome reports are received [32].

## 4. Discussion

This rapid review has identified several matters relating to the roles and responsibilities of the pharmacist workforce supporting older people living in a community setting with palliative care needs. Despite diversity in the health care systems across the USA, Canada, UK, and Australia from where the studies were generated, similar themes across the literature were observed.

Reviewed papers predominantly focused on care provided by the pharmacist for people receiving RAC services; only a few examined the role of pharmacists with older people receiving HC services. This may be explained by the complex health and social care needs of people receiving RAC services. Services established for delivery of RAC are also likely to have more formal systems and processes in place as a result of contractual arrangements, making this aspect of care easier to review and assess. In contrast, HC service provision is less visible due to informal processes and relationships between individuals.

The findings observed in this rapid review suggest that a pharmacist’s role (in developed countries) continues to evolve beyond their traditional medication dispensing responsibilities, with pharmacists stepping away from the dispensary and gaining larger significance in RAC services and General Medical Practices. A focus on the clinical role of pharmacists when working with older people in the community—including guidance on deprescribing, monitoring of medicines use, and detecting adverse drug events—is particularly important considering the growing prevalence of age-related multimorbidity resulting in polypharmacy and the increasing number of older populations receiving RAC and HC services.

In Australia, existing government-funded programs support pharmacists conducting clinical reviews, including: MedsChecks, home medicines reviews (HMRs), and residential medication management reviews (RMMRs) [12]. Such programs aim to prevent adverse drug reactions, improving clinical care and reducing unnecessary usage of medicines [34]. As well as providing direct clinical outcomes, medication reviews improve communication between pharmacists and the multidisciplinary team. As such, existing government funding levers may provide a useful instrument to involve pharmacists within aged care organisations. In 2018, the Australian government funded almost half a million medication reviews across the entire population, with 22% as RMMRs, 16% as HMRs, and the remaining 62% as MedsChecks [35]. With over 3.6 million Australians aged 65 years or more [36], there is a significant capacity for expanding the number of funded medication reviews by accredited pharmacists in older people each year. The Australian Government has recently relaxed the referral process for HMRs and RMMRs, permitting any Medical Practitioner to refer a patient for a medication review [37,38].

This review also identified organizational wide benefits of involving pharmacists from involvement on MACs to the conducting of audits or guideline reviews. While articles discussing this broader role for pharmacists were limited to organisations providing RAC services, NGOs such as those providing HC services could learn from the RAC experience, particularly in light of the Royal Commission into Aged Care [39]. The Royal Commission has identified several concerns—including poorly executed palliative care and excessive use of sedatives—where pharmacists could play critical roles in the development of safeguards, ensuring good medication management for all older people [40]. Furthermore, the soft skills inherent in the pharmacist workforce may augment the more formal processes that support good management of medicines within organisations. The Pharmaceutical Society of Australia (PSA) National Competency Standards Framework for Pharmacists In Australia supports pharmacists’ role in multidisciplinary teams by saying pharmacists “show a commitment to interprofessional practice” [41]. Consideration of the diverse role of the pharmacist and their broader benefits to the multidisciplinary aged care workforce, including GPs and those delivering HC services, should be studied.

The pharmacist’s role within the multidisciplinary team—supporting the care of older people—has been established: contributing to the improvement in health outcomes by working with others to provide medication management in older people who take multiple medications within a context of complex health care needs [42,43,44]. Roles of pharmacists within the context of multidisciplinary palliative care may strengthen the evidence base for good medicines management where RAC and HC services are delivered [45].

## Implications for Policy and Practice in Aged Care

Health care services provided to older Australians are delivered by multiple providers across primary, secondary, and tertiary health care services. These services are often fragmented, with poor information sharing at points of transition. In addition, polypharmacy is inherent in the older population, making them more vulnerable to several risks, including adverse drug reactions and drug interactions. As such, pharmacists have a significant opportunity to contribute to and ensure appropriate and timely provision of medication and ensure that medication advice is available for all older Australians [46]. There are growing calls for the expansion of pharmacist roles beyond dispensing and clinical reviews while streamlining funding pathways through the mechanism of pharmacist access to the MBS and PBS reimbursements [47]. These expanded roles may serve to free up valuable GP time to manage more complex or acute medical problems, leading to a reduction in delays in essential end of life care for all older Australians [47].

## 5. Limitations

This rapid review was a resource constraint and time bound analysis. We applied a search strategy involving only three databases, focusing on indexed English language literature. Due to the streamlined search approach, relevant papers indexed in other databases and non-English literature would have been missed. The search approach adopted in this rapid review, however, is in line with existing rapid reviews that reported to have searched a minimum of two databases to retrieve literature and synthesise data [48].

The rapid review only located papers of level III and level V evidence. There were no papers graded at Level I, suggesting that scientifically strong papers in this area may be lacking. The lack of papers classified into the Level I category may also have been due to our specific research question and search strategies. The review question applied in this study sought a snapshot of the evidence exploring pharmacist’s role in supporting the older population with palliative care needs. Therefore, intervention studies assessing the effectiveness of the roles of community pharmacists within the context of multidisciplinary palliative care may have been missed due to our streamlined research question and search strategies.

Further, this review resulted into relevant papers being sourced predominantly from developed countries, including the USA, Canada, UK, and Australia. As such, the health care settings in developing and less developed countries may not reflect the same health system structure and practices. In other countries, the community pharmacist role may not be as well established and they may not have the same roles and responsibilities identified in this review. Hence, the results generated from this review may not be generalisable in developing and less developed health care settings.

## 6. Conclusions

The roles and responsibilities of community pharmacists continue to evolve. The multifaceted role of pharmacists presents an opportunity to provide comprehensive medicines management for the older population at the end of their life. There is scope within the current health care system to increase organisational support for pharmacists working with older populations in aged care organisations delivering RAC or HC services. This is likely to facilitate better management of medication and improved care on discharge from the acute sector for older people with palliative care needs. Further studies should aim to build the level of evidence relating to the effectiveness of pharmacist roles in supporting people living with palliative care needs in the community.

## Figures and Tables

**Table 1:** Criteria for title and abstract screening, and full-text review of the included papers.

No.	Criterion	Description
1.	Population of interest	Pharmacists practising predominantly in dispensing or non-dispensing role.
2.	Settings of interest	Community setting comprising dispensing pharmacy, general medical practice, residential aged care facility, Aboriginal health services, and peoples’ own home.
3.	Phenomenon of interest	Roles and responsibilities of community pharmacists supporting older people aged 65 years and over and their carer living in the community with palliative care needs.
4.	Types of studies	Quantitative or qualitative studies, including peer-reviewed journal articles and grey literature documents. Studies were selected if they reported one or more of the inclusion criteria (i.e., 1–3) outlined above.

**Table 2:** Evidence type used in appraising the quality of the evidence of included papers.

Level Type	Description
Level I	Experimental, randomized controlled trial (RCT), systematic review RTCs with or without meta-analysis
Level II	Quasi-experimental studies, systematic review of a combination of RCTs and quasi-experimental studies, or quasi-experimental studies only, with or without meta-analysis
Level III	Nonexperimental, systematic review of RCTs, quasi-experimental with/without meta-analysis, qualitative, qualitative systematic review with/without meta-synthesis
Level IV	Respected authorities’ opinions, nationally recognized expert committee or consensus panel reports based on scientific evidence
Level V	Literature reviews, quality improvement, program evaluation, financial evaluation, case reports, nationally recognized expert(s) opinion based on experiential evidence

**Table 3:** Characteristics, level of evidence, count of data elements, and summary description of selected papers.

Author, Year	Title	Study Design	Setting	Country	Level of Evidence	Count of Data Elements	Summary Description
Burns, 2014 [20]	New horizons in care home medicine	Systematic review of experimental, quasi experimental, and non-experimental studies	Residential Aged Care Home (RACH)	UK	Level III	8	Reviews role of RACH staff including pharmacists in integrated models of care supporting better outcomes for older people.
Crecelius, 2006 [21]	Pain Control: No Time to Rest on Our Laurels	Expert opinion	RACH	USA	Level V	7	Provides expert commentary on pain management for older people living in RACH environments
Disalvo, 2019 [22]	Pharmacists’ perspectives on medication reviews for long-term care residents with advanced dementia: a qualitative study	Qualitative study using semi-structured interview	RACH	Australia	Level III	29	Explores pharmacist perspectives of the Australian Government funded residential medication management review and its role improving the quality and safety of prescribing for people with advanced dementia.
Elliott, 2016 [23]	Medicines Management, Medication Errors and Adverse Medication Events in Older People Referred to a Community Nursing Service: A Retrospective Observational Study	Retrospective records audit and telephone interview	Home Care	Australia	Level III	12	Explores the characteristics of older people referred for medicines management support, type of support provided, medication errors, and Adverse Drug Reactions.
Hays, 1984 [24]	Home Care of the Frail Elderly And the Terminally Ill	Expert opinion	Home Care	UK	Level V	5	Discusses general principles of managing elderly and terminally ill patients in a home environment.
Kuruvilla, 2018 [25]	Medication management for community palliative care patients and the role of a specialist palliative care pharmacist: A qualitative exploration of consumer and health care professional perspectives	Qualitative study using focus group	Both RACH and Home Care	Australia	Level III	20	Explores the gaps in the current model of community palliative care services on medication management and the role of a pharmacist in addressing these.
LaMantia, 2010 [26]	Interventions to Improve Transitional Care Between Nursing Homes and Hospitals: A Systematic Review	Systematic review of experimental, quasi experimental, and non-experimental studies	RACH	USA	Level III	7	Identifies and evaluates interventions to improve the communication of accurate and appropriate medication lists and advance directives for older people who transition between a RACH and a hospital.
Martin, 2011 [27]	There’s No Place Like Home: A Pharmacist Fills the Need	Case report	Home Care	USA	Level V	14	Describes the practice of a pharmacist working with older people receiving home care.
Meade, 2006 [28]	Innovative Services for Assisted Living, Hospice, and the Community	Case report	Both RACH and Home Care	USA	Level V	29	Describes the practice of a pharmacist who provides medication management services to older people living in a RACH or receiving home care.
Noyce, 1990 [29]	Intramural and extramural health care in the United Kingdom	Expert opinion	Both RACH and Home Care	UK	Level V	8	Describes the factors that determine whether health care in the United Kingdom is provided in hospital, at home, or through intermediate or shared care arrangements.
Prukowski, 2017 [30]	The DE-PHARM Project: A Pharmacist-Driven Deprescribing Initiative in a Nursing Facility	Quality improvement intervention study	RACH	USA	Level V	10	Assesses the acceptance of recommendations from the pharmacist to the primary care team regarding the discontinuation of medications used for the management of comorbid diagnoses.
Tait, 2017 [33]	Improving community access to terminal phase medicines through the implementation of a “Core Medicines List” in South Australian community pharmacies	Qualitative study using repeat survey	Both RACH and Home Care	Australia	Level III	14	Identifies changes in community access to medicines for managing symptoms in the terminal phase following the development of a “Core Medicines List”.
Tamura, 2012 [31]	Outcomes of Polypharmacy in Nursing Home Residents	Comprehensive literature review	RACH	USA	Level V	13	Reviews the outcomes of polypharmacy in RACHs.
Tija, 2013 [32]	Studies to Reduce Unnecessary Medication Use in Frail Older Adults: A Systematic Review	Systematic review of experimental, quasi experimental, and non-experimental studies	Both RACH and Home Care	USA	Level III	20	Identifies interventions that reduce the use of unnecessary medications in frail older adults and patients approaching end of life.

**Table 4:** Taxonomy of the themes identified and illustrated with key roles and responsibilities of the pharmacists that emerged from the literature synthesis.

Theme (n = 3)	% (n) of Data Elements	Definition	Domain (n = 8)	Meaning Unit (n = 37)
Type of care delivery	72% (n = 140)	Pharmacists support the medicines management of people living with palliative care needs directly with the patients themselves and indirectly by improving the performance of the organisation.	Clinical review	Reconciling medications; Deprescribing; Guiding the adjustment of medication doses; Identifying medication related problems; Assessing appropriateness and safety of prescribed medications;
			Supply of medicines	Stocking subcutaneous injections; Dispensing; Returning of unwanted medicines; Delivering Medicines to the home; Supplying medicines to a residential aged care home; Offering a dose administration aid service; Providing medicines information; Counselling and educational intervention
			Clinical governance	Participating on Medicines Advisory Committees in residential aged care home; Educating nursing workforce including carers; Auditing of medications; Developing policies and guidelines
Work setting of the pharmacist	20% (n = 40)	Pharmacists collaborate with multidisciplinary workforce to achieve optimal results in patient care.	Community Pharmacy	Clarifying prescriptions with prescribers; Improving access to subcutaneous medicines; Participating in case conferences; Discussing medication review findings
			Residential Aged Care Homes	Reviewing medicines on admission; Participating in multidisciplinary medication reviews; Participating in case conferences; Understanding patient’s goals of care; Supplying medicines to RACH imprest stock
			General Medical Practice	Offering a clinical resource; Providing medicines information; Improving efficiency of medication reviews
Supportive professional and personal characteristics as soft skills	8% (n = 16)	Pharmacists use soft skills in their role to assist and provide support to patients with their medication management.	Soft skills in supporting person-centred care	Advocating; Following-up
			Soft skills in dealing with clinician prescribers	Framing of recommendations; Building trusting relationships; Developing creative communication approaches; Demonstrating a positive and helpful attitude; Communicating in a clear and honest manner; Facilitating referrals

## Supplementary Materials

The following are available online at https://www.mdpi.com/2226-4787/8/3/143/s1, Table S1. Database(s): Ovid MEDLINE(R) and Epub Ahead of Print, In-Process & Other Non-Indexed Citations, Daily and Versions(R) (Search conducted: 19 July 2019). Table S2. Database: Cinahl (Ebsco) (Search conducted: 23 July 2019). Table S3. Database: Scopus (Search conducted: 23 July 2019). Table S4. Completed PRISMA checklist.
