# Supplementary material for: The Roles and Responsibilities of Community Pharmacists Supporting Older People with Palliative Care Needs: A Rapid Review of the Literature
Source: Pharmacy (Basel). 2020 Aug 12;8(3):143. doi: 10.3390/pharmacy8030143 (PMC7558267; doi:10.3390/pharmacy8030143)
Supplement: Supplementary file 1 [file pharmacy-08-00143-s001.pdf]

The roles and responsibilities of community pharmacists supporting older populations with palliative care needs: a rapid review of the literature

Paul Tait, Amal Chakraborty, and Jennifer Tieman

Supplementary File: Peer reviewed search strategy and PRISMA checklist

Table S1. Database(s): Ovid MEDLINE(R) and Epub Ahead of Print, In-Process & Other Non-Indexed Citations, Daily and Versions(R) (Search conducted: 19 July 2019).

Search #	Query
1	exp advance care planning/
2	exp attitude to death/
3	exp bereavement/
4	death/
5	life support care/
6	palliative care/
7	exp terminal care/
8	terminally ill/
9	(palliat* or advance care plan* or advance directive* or bereavement).tw,kw.
10	(terminal care or terminally ill).tw,kw.
11	end of life.tw,kw.
12	or/1-11
13	(homes for the aged or residential aged care or aged care facilit*).mp. [mp=title, abstract, original title, name of substance word, subject heading word, floating sub-heading word, keyword heading word, organism supplementary concept word, protocol supplementary concept word, rare disease supplementary concept word, unique identifier, synonyms]
14	(long term care or longterm care or home* or homecare or homebound or community).mp. [mp=title, abstract, original title, name of substance word, subject heading word, floating sub-heading word, keyword heading word, organism supplementary concept word, protocol supplementary concept word, rare disease supplementary concept word, unique identifier, synonyms]
15	(aged adj ("65" or "70" or "75" or "80" or "85")).tw,kw.
16	aged/ or "aged, 80 and over"/ or frail elderly/ or exp dementia/
17	(elder* or geriatric* or gerontolog* or old age* or grandparent* or retire* or pensioner* or later life or aged care or dementia or alzheimer*).mp.
18	((old* or aged or aging) adj (person or people* or adult* or resident* or population* or men* or women* or male* or female* or patient*)).tw,kw.
19	15 or 16 or 17 or 18
20	14 and 19
21	13 or 20
22	(pharmacist* or pharmacy or drugstore* or drug store* or dispensary).mp.
23	((prescribe* or review* or prescription* or supply or provide or provision or dispense or availability) adj1 (medication* or medicine* or drug*)).mp. [mp=title, abstract, original title, name of substance word, subject heading word, floating sub-heading word, keyword

Search #	Query
	heading word, organism supplementary concept word, protocol supplementary concept word, rare disease supplementary concept word, unique identifier, synonyms]
24	22 or 23
25	12 and 21 and 24
26	limit 25 to english language

Table S2. Database: Cinahl (Ebsco) (Search conducted: 23 July 2019).

Search #	Query
S1	(MH "Palliative Care") OR (MH "Hospice Care") OR (MH "Terminal Care") OR (MH "Hospice and Palliative Nursing")
S2	(MH "Bereavement") OR (MH "Attitude to Death")
S3	((MH "Terminally Ill Patients") OR (MH "Advance Care Planning") OR (MH "Living Wills") OR (MH "Advance Directives")
S4	TI (palliat* or "advance care plan*" or "advance directive*" or bereavement) OR AB (palliat* or "advance care plan*" or "advance directive*" or bereavement)
S5	TI ("terminal care" or "terminally ill" or "end of life") OR AB ("terminal care" or "terminally ill" or "end of life")
S6	S1 OR S2 OR S3 OR S4 OR S5
S7	(MH "Aged") OR (MH "Frail Elderly") OR (MH "Aged, 80 and Over")
S8	(MH "Dementia")
S9	TI (elder* or geriatric* or gerontolog* or "old age*" or grandparent* or retire* or pensioner* or "later life" or "aged care" or dementia or alzheimer*) OR AB (elder* or geriatric* or gerontolog* or "old age*" or grandparent* or retire* or pensioner* or "later life" or "aged care" or dementia or alzheimer*)
S10	TI ((old* or aged or aging) W1 (person or people* or adult* or resident* or population* or men* or women* or male* or female* or patient*)) OR AB ((old* or aged or aging) W1 (person or people* or adult* or resident* or population* or men* or women* or male* or female* or patient*))
S11	S7 OR S8 OR S9 OR S10
S12	"long term care" or "longterm care" or home* or homecare or homebound or community
S13	"residential aged care" OR "aged care facilit*"
S14	S11 AND S12
S15	S13 OR S14
S16	pharmacist* or pharmacy or chemist or chemists or drugstore* or drug store* or dispensary

S17	((prescribe* or review* or prescription* or supply or provide or provision or dispense or availability) N1 (medication* or medicine* or drug*))
S18	S16 OR S17
S19	S6 AND S15 AND S18

Table S3. Database: Scopus (Search conducted: 23 July 2019).

Search terms
(((TITLE-ABS-KEY ("long term care" OR "longterm care" OR home* OR homecare OR homebound OR community)) AND ((TITLE-ABS-KEY (elder* OR geriatric* OR gerontolog* OR "old age*" OR grandparent* OR retire* OR pensioner* OR "later life" OR "aged care" OR dementia OR alzheimer*)) OR ((TITLE-ABS-KEY ((old* OR aged OR aging) PRE/1 (person OR people* OR adult* OR resident* OR population* OR men* OR women* OR male* OR female* OR patient*)) OR TITLE-ABS-KEY (aged PRE/1 ("65" OR "70" OR "75" OR "80" OR "85")))))) OR (TITLE-ABS-KEY ("residential aged care" OR "aged care facilit*"))) AND (TITLE-ABS-KEY ((palliat* OR "advance care plan*" OR "advance directive*" OR bereavement OR "terminal care" OR hospice* OR "terminally ill" OR "end of life"))) AND ((TITLE-ABS-KEY ((prescribe* OR review* OR prescription* OR supply OR provide OR provision OR dispense OR availability) W/1 (medication* OR medicine* OR drug*)) OR TITLE-ABS-KEY (pharmacist* OR pharmacy OR drugstore* OR drug AND store* OR dispensary)))
Search limited to English

Table S4. Completed PRISMA checklist.

**Section/topic**	#	**Checklist item**	**Reported on page #**
**TITLE**			
Title	1	Identify the report as a systematic review, meta-analysis, or both.	p. 1
**ABSTRACT**			
Structured summary	2	Provide a structured summary including, as applicable: background; objectives; data sources; study eligibility criteria, participants, and interventions; study appraisal and synthesis methods; results; limitations; conclusions and implications of key findings; systematic review registration number.	p. 1
**INTRODUCTION**			
Rationale	3	Describe the rationale for the review in the context of what is already known.	pp. 1-2
Objectives	4	Provide an explicit statement of questions being addressed with reference to participants, interventions, comparisons, outcomes, and study design (PICOS).	p. 2
**METHODS**			
Protocol and registration	5	Indicate if a review protocol exists, if and where it can be accessed (e.g., Web address), and, if available, provide registration information including registration number.	Not applicable
Eligibility criteria	6	Specify study characteristics (e.g., PICOS, length of follow-up) and report characteristics (e.g., years considered, language, publication status) used as criteria for eligibility, giving rationale.	p. 3; Table 1
Information sources	7	Describe all information sources (e.g., databases with dates of coverage, contact with study authors to identify additional studies) in the search and date last searched.	pp. 2
Search	8	Present full electronic search strategy for at least one database, including any limits used, such that it could be repeated.	pp. 2; Supplementary Tables S1-3
Study selection	9	State the process for selecting studies (i.e., screening, eligibility, included in systematic review, and, if applicable, included in the meta-analysis).	p. 2-3
Data collection process	10	Describe method of data extraction from reports (e.g., piloted forms, independently, in duplicate) and any processes for obtaining and confirming data from investigators.	p. 3
Data items	11	List and define all variables for which data were sought (e.g., PICOS, funding sources) and any assumptions and simplifications made.	p. 3

Section/topic	#	Checklist item	Reported on page #
Risk of bias in individual studies	12	Describe methods used for assessing risk of bias of individual studies (including specification of whether this was done at the study or outcome level), and how this information is to be used in any data synthesis.	Not applicable
Summary measures	13	State the principal summary measures (e.g., risk ratio, difference in means).	Not applicable
Synthesis of results	14	Describe the methods of handling data and combining results of studies, if done, including measures of consistency (e.g., I2) for each meta-analysis.	pp. 4, Systematic text condensation
Risk of bias across studies	15	Specify any assessment of risk of bias that may affect the cumulative evidence (e.g., publication bias, selective reporting within studies).	Not applicable
Additional analyses	16	Describe methods of additional analyses (e.g., sensitivity or subgroup analyses, meta-regression), if done, indicating which were pre-specified.	p. 3-4, Table 2, Level of evidence
**RESULTS**			
Study selection	17	Give numbers of studies screened, assessed for eligibility, and included in the review, with reasons for exclusions at each stage, ideally with a flow diagram.	p. 4-5; Figure 1
Study characteristics	18	For each study, present characteristics for which data were extracted (e.g., study size, PICOS, follow-up period) and provide the citations.	p. 4-7; Table 3
Risk of bias within studies	19	Present data on risk of bias of each study and, if available, any outcome level assessment (see item 12).	Not applicable
Results of individual studies	20	For all outcomes considered (benefits or harms), present, for each study: (a) simple summary data for each intervention group (b) effect estimates and confidence intervals, ideally with a forest plot.	pp. 4-7; Table 3
Synthesis of results	21	Present results of each meta-analysis done, including confidence intervals and measures of consistency.	pp. 8-11; Table 4, Systematic text condensation
Risk of bias across studies	22	Present results of any assessment of risk of bias across studies (see Item 15).	Not applicable
Additional analysis	23	Give results of additional analyses, if done (e.g., sensitivity or subgroup analyses, meta-regression [see Item 16]).	p. 4-7, Table 3, Level of evidence
**DISCUSSION**			
Summary of evidence	24	Summarize the main findings including the strength of evidence for each main outcome; consider their relevance to key groups (e.g., healthcare providers, users, and policy makers).	p. 11-12
Limitations	25	Discuss limitations at study and outcome level (e.g., risk of bias), and at review-level (e.g., incomplete retrieval of identified research, reporting bias).	p. 12

Section/topic	#	Checklist item	Reported on page #
Conclusions	26	Provide a general interpretation of the results in the context of other evidence, and implications for future research.	p. 13
**FUNDING**			
Funding	27	Describe sources of funding for the systematic review and other support (e.g., supply of data); role of funders for the systematic review.	p. 13

From: Moher D, Liberati A, Tetzlaff J, Altman DG, The PRISMA Group (2009). Preferred Reporting Items for Systematic Reviews and Meta-Analyses: The PRISMA Statement. PLoS Med 6(7): e1000097. doi:10.1371/journal.pmed1000097
For more information, visit: www.prisma-statement.org.
